# Comparative analysis of follicular cell- derived thyroid carcinoma: assessing the impact of high-grade features in an advanced disease cohort

**DOI:** 10.1007/s00428-025-04109-2

**Published:** 2025-05-02

**Authors:** Mariana Caldeira, Sule Canberk, Sofia Macedo, Miguel Melo, Valdemar Máximo, Paula Soares

**Affiliations:** 1 Faculty of Medicine of the University of Porto (FMUP), Alameda Professor Hernâni Monteiro, 4200 - 319 Porto, Portugal; 2 Cancer Signalling and Metabolism Group, Instituto de Investigação e Inovação em Saúde (i3S), University of Porto, Rua Alfredo Allen 208, 4200 - 135 Porto, Portugal; 3 Department of Pathology, Faculty of Medicine of the University of Porto (FMUP), Alameda Prof. Hernâni Monteiro, 4200 - 319 Porto, Portugal; 4 Institute of Molecular Pathology and Immunology of the University of Porto (Ipatimup), Rua Júlio Amaral de Carvalho 45, 4200 - 135 Porto, Portugal; 5 Department of Endocrinology, Diabetes and Metabolism, Centro Hospitalar E Universitário de Coimbra, Coimbra, Portugal; 6 Medical Faculty, University of Coimbra, Coimbra, Portugal

**Keywords:** Thyroid carcinoma, Differentiated thyroid carcinoma, Differentiated high-grade thyroid carcinoma, Poorly differentiated thyroid carcinoma, Advanced thyroid carcinoma

## Abstract

The 5th edition of the WHO Classification of Tumors of Endocrine Organs introduced the term *Differentiated High-Grade Thyroid Carcinoma (DHGTC)* to identify cases of differentiated follicular cell-derived thyroid carcinomas (DFCDTC) with a worse prognosis. This study aimed to determine the frequency and clinicopathological features of DHGTC within a cohort of advanced follicular cell-derived thyroid carcinomas (AdvTC) and compare them to non-high-grade DFCDTC (non-HGDTC) and poorly differentiated thyroid carcinoma (PDTC). A retrospective analysis was conducted on 138 patients with AdvTC who underwent total thyroidectomy followed by radioactive iodine therapy (131I). DHGTC was identified in 15.9% of the cases (22/138), showing a higher prevalence in this selected cohort of AdvTC compared to other studies. Compared to non-HGDTC, DHGTC was significantly associated with adverse clinicopathological features, including age ranges ≤ 18 and ≥ 55 years, presence of distant and synchronous metastasis, larger tumor size (> 2 cm), tall-cell subtype of papillary thyroid carcinoma, higher mitotic index (≥ 5/2 mm^2^), tumor necrosis, angioinvasion, higher AJCC 8th edition pT stage (pT3/T4), and more frequent administration of additional therapies, such as tyrosine kinase inhibitors. In comparison to PDTC, DHGTC displayed lower median tumor size, less frequent tumor necrosis, and a higher mitotic count. Independent prognostic factors for worse DSS in the entire cohort were age ≥ 55 years (HR = 19.625, *p* = 0.005) and male sex (HR = 7.441, *p* = 0.029). DHGTC cases consistently demonstrated worse clinical outcomes compared to non-HGDTC, including lower survival rates and higher persistence of disease at the end of follow-up. Our results validate the inclusion of DHGTC as a distinct high-grade subgroup within follicular cell-derived thyroid carcinomas, as proposed by the 5th WHO classification. DHGTC exhibits aggressive clinicopathological features and poor outcomes, supporting its relevance in clinical risk stratification and therapeutic decision-making.

**Supplementary information:**

The online version contains supplementary material available at 10.1007/s00428-025-04109-2.

## Introduction

Differentiated follicular cell-derived thyroid carcinoma (DFCDTC), which includes papillary (PTC), follicular (FTC), and oncocytic (OCA) histotypes, is typically associated with a less aggressive clinical course and low mortality rate. However, a subset of DFCDTC exhibits aggressive behavior, such as resistance to radioactive iodine (RAI) therapy and recurrence/metastasis [1]. While the clinical prognosis of FTC and OCA can be more predictable based on their subtypes—such as minimally invasive, encapsulated angioinvasive, and widely invasive—predicting the aggressiveness of papillary thyroid carcinoma (PTC) is more challenging [2]. This difficulty arises because the cellular morphology and architectural patterns used to classify PTC into aggressive and non-aggressive subtypes do not always reliably correlate with clinical outcomes [3].

In the 2022 World Health Organization (WHO) classification of thyroid neoplasms [4], evidence from the Memorial Sloan Kettering Cancer Center’s (MSKCC) criteria [5] and the Turin consensus’ criteria [6] was used to identify high-grade follicular cell-derived non-anaplastic thyroid carcinoma. This classification recognizes two subtypes: the well-documented poorly differentiated thyroid carcinoma (PDTC) and the newly defined differentiated high-grade thyroid carcinoma (DHGTC) [7].

PDTC is known for its aggressive clinical course [8], whereas DHGTC, encompassing high-grade feature subtypes of PTC, FTC, and OCA, represents an emergent concept aimed to integrate a prognostic perspective in the histologic classification by identifying tumors at an increased risk of aggressive clinical outcomes [9]. To classify a tumor as DHGTC it needs to have papillary, follicular or solid growth pattern; any nuclear cytology; absence of anaplastic features and one of the following two features: mitotic count ≥ 5 per 2 mm^2^ and/or tumor necrosis [9].

Before the introduction of the DHGTC term, these criteria have already been in use (mitotic count ≥ 3 or 5 per 2 mm^2^ and/or tumor necrosis) and created new concepts: “PTC With High-Grade Features” [10]; “PDTC according to MSKCC criteria” [11]; or “HGTC-nonPDTC” (High-Grade Follicular Cell-Derived Non-Anaplastic Thyroid Carcinoma that did not fulfill Turin’s consensus criteria for PDTC) [12], which were shown to distinguished worse clinical outcomes and more aggressive clinicopathological features and motivated this change in the the 5 th WHO Classification.

After the introduction of the new classification, more articles are being published [13–16, 16, 17], with interesting results, which highlight a need to continue developing more and larger studies to fully elucidate their clinical and pathological characteristics.

Our study focuses on advanced thyroid carcinoma (AdvTC), to elucidate the clinicopathological and molecular attributes of DHGTC within a cohort exhibiting poor prognosis features, such as a high rate of recurrence and metastasis. To effectively contextualize our comparison, it is imperative to define what constitutes “AdvTC” within the scope of this study, as there is currently no widely accepted definition across all disciplines [1]. AdvTC typically refers to tumors which exhibit extensive local invasion, distant metastasis, or resistance to conventional therapies, including RAI. In our cohort, the definition for AdvTC aligns with these characteristics, further specified by a high recurrence rate and frequent development of metastasis. This definition adheres to the 2022 guidelines of the American Head and Neck Society Endocrine Surgery Section (AHNS) and the International Thyroid Oncology Group (ITOG), providing a robust framework for the selection and analysis of our cohort [18].

In sum, the main aims of the study were: (i) to describe the clinical behavior, pathological features and prognostic factors of a cohort of patients with AdvTC; (ii) to review the cases according to the 5 th edition of WHO’s criteria, define the prevalence of DHGTC in the series, and evaluate its clinical behavior and prognostic factors and (iii) to assess the clinicopathological differences between DHGTC vs non-HGDTC and DHGTC vs PDTC.

## Materials and methods

### Tumor samples and data collection

A total of 138 formalin-fixed, paraffin-embedded (FFPE) samples of human thyroid tissue from patients followed in Centro Hospitalar e Universitário de Coimbra, Portugal, kept at the biobank of the Institute of Molecular Pathology and Immunology of the University of Porto were included in our series. Clinicopathological data was retrieved from clinical records by MM. The patient samples were obtained with informed consent and analyzed based on the local data protection laws. The study approval was given by the Ethics Committee of the Faculty of Medicine of the University of Coimbra (n° 1309) and regulated in compliance with national ethical standards (Law n° 12/2005) and the Helsinki Declaration.

### Characteristics of the cohort and clinicopathological review

The patients were diagnosed with thyroid cancer between 1971 and 2012 and underwent total thyroidectomy, followed by therapy with radioactive iodine (131I). The cohort’s characteristics were reevaluated to apply the 10 criteria of the consensus statement on advanced thyroid cancer from AHNS and ITOG (Supplementary Table S1).

Synchronous metastases were defined as metastasis identified within 6 months after the diagnosis of the primary cancer [19].

An endocrine pathologist (SC) reviewed the histology slides, applying the criteria of the 5 th edition of WHO’s classification with blindness to the clinical outcomes. None of the 9 cases classified as Invasive Encapsulated Follicular Variant of PTC (IEFVPTC) cases were high-grade and, despite knowing that the 5 th WHO edition recognizes IEFVPTC as a distinct entity rather than a PTC subtype, to simplify statistical analysis, all IEFVPTC cases were classified under PTC in our study.

**Fig. 1:**
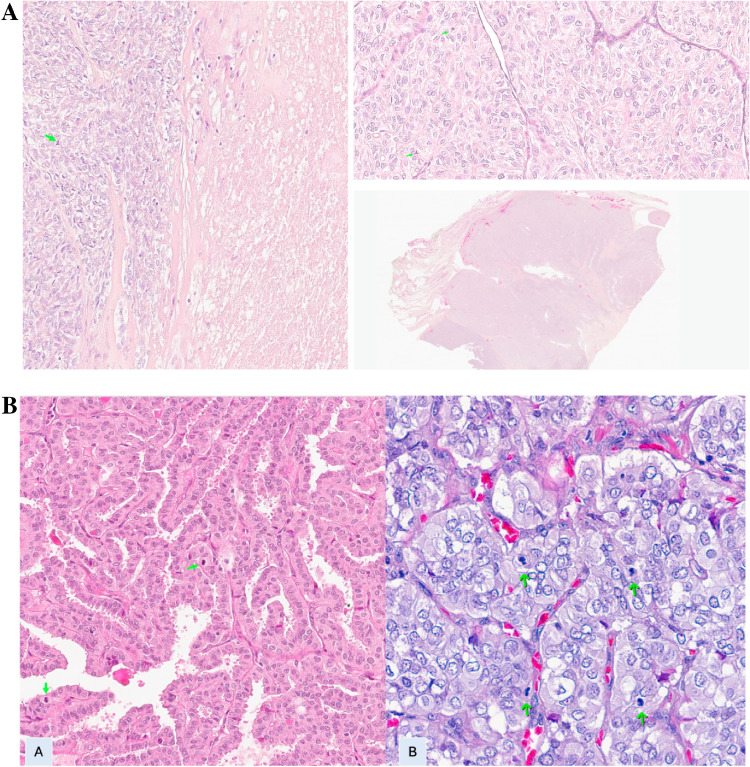
**A** Examples of readily identifiable mitoses (A and B green arrows) in PDTC in 400 × magnification. Area of tumor necrosis in PDTC (C and D) in 20 × magnification. **B** Examples of readily identifiable mitoses (A and B green arrows) in PTC, oncocytic subtype and infiltrative follicular subtype in 20 and 40 × magnification

DHGTC was classified according to the criteria of 5 th edition of the WHO classification of thyroid neoplasms, with mitosis given per 2 mm.^2^ and counted in the most mitotically active"hotspot"areas [2, 20]. Tumor necrosis was characterized by specific signs of irreversible cellular damage. These included the presence of nuclear debris and the formation of distinct ghost cells or nuclear outlines, clearly demarcated to indicate cell death (Fig. 1A and 1B). Data from genetic characterization of the tumors was obtained in previous works of the group which included part of the cases included in this series: In our study of 138 cases, mutation analysis was performed in 123 cases for *BRAF* V600E, 91 cases for *NRAS* Q61R, and 108 cases for the *TERT* promoter (Table 1) [21, 22].

**Table 1:** Most relevant clinicopathologic characteristics of 138 patients with primarily resected advanced thyroid cancer

	All [*n* = 138]	Non-HGDTC [*n* = 106]	DHGTC [*n* = 22]	PDTC [*n* = 10]	*P*value (DHGTC vs non-HGDTC)	*P*value (DHGTC vs PDTC)
Clinicopathological parameters						
Age (years) [*n* = 137]						
Median (range) [1 st quartile; 3rd quartile]	45 (13–81) [34;57,5]	44,5 (17–74) [34;56]	48 (13–81) [31; 71,5]	53 (22–69) [42; 65,5]	NS	NS
≤ 18	4 (3,1%)	1 (0,9%)	3 (13,6%)	0 (0%)	0,016	
18–55	91 (65,9%)	77 (72,6%)	8 (38,1%)	6 (60%)	< 0,001	NS
≥ 55	42 (30,4%)	28 (26,4%)	10 (47,6%)	4 (40%)	< 0,001	
Sex						
Female	108 (78,3%)	16 (72,7%)	86 (81,1%)	6 (60%)	NS	NS
Male	30 (21,7%)	6 (27,3%)	20 (18,9%)	4 (40%)		
Distant metastasis	36 (26,1%)	19 (17,9%)	10 (45,5%)	7 (70%)	0,008	NS
Types of distant metastasis [*n* = 36] #						
Bone	13 (9,4%)	8 (7,5%)	3 (13,6%)	2 (20%)	NS	NS
Lung	26 (18,8%)	12 (11,3%)	8 (36,3%)	6 (20%)	0,015	
Brain	2 (1,4%)	0 (0%)	1 (4,5%)	1 (10%)	NS	
Kidney	1 (0,7%)	0 (0%)	1 (4,5%)	0 (0%)	NS	
Synchronous metastasis [*n* = 127]	27 (21,3%)	6 (35,3%)	14 (14%)	7 (70%)	0,042	NS
Tumor size (cm) [*n* = 125]						
Median (range) [1 st quartile; 3rd quartile]	2,65 (0,5–9)	2,3 (0,5–9) [1,5; 2,7]	3,51 (0,8–8) [2,0; 4,8]	5,0 (2,5–8) [2,0; 4,4]	0,007	0,023
≤ 2 cm	67 (53,6%)	62 (61,4%)	5 (31,3%)	0 (0%)	0,023	NS
> 2 cm	58 (46,4%)	39 (38,6%)	11 (68,7%)	8 (100%)		
Main diagnosis						
Classical subtype of PTC	40 (29%)	35 (33%)	5 (22,7%)	NA	NS	NA
Hobnail subtype of PTC	6 (4,3%)	5 (4,7%)	1 (4,5%)	NA	NS	NA
Invasive encapsulated follicular subtype of PTC	9 (6,5%)	9 (8,5%)	0 (0%)	NA	NS	NA
Infiltrative follicular subtype of PTC	27 (19,6%)	24 (22,6%)	3 (13,6%)	NA	NS	NA
Oncocytic subtype of PTC	9 (6,5%)	9 (8,5%)	0 (0%)	NA	NS	NA
Solid subtype of PTC	6 (4,3%)	4 (3,8%)	2 (9,1%)	NA	NS	NA
Tall-cell subtype of PTC	17 (12,3)	8 (7,5%)	9 (37,5%)	NA	< 0,001	NA
Warthin-like subtype of PTC	7 (5,1%)	7 (6,6%)	0 (0%)	NA	NS	NA
Oncocytic carcinoma of the thyroid	2 (1,4%)	1 (0,9%)	1 (4,5%)	NA	NS	NA
Follicular thyroid carcinoma	5 (3,6%)	4 (3,8%)	1 (4,5%)	NA	NS	NA
Poorly differentiated thyroid carcinoma	10 (7,2%)	NA	NA	10 (100%)	NA	NA
Mitotic index (per 2 mm2)						
Median (range) [1 st quartile; 3rd quartile]	2 (0–8) [1;4]	1 (0–4) [1;2]	6 (5–8)] [5; 6,25]	4,5 (3–8) [3,75; 5,25]	< 0,001	0,009
< 5	111 (80,4%)	106 (100%)	0 (0%)	5 (50%)	< 0,001	< 0,001
≥ 5	27 (19,6%)	0 (0%)	22 (100%)	5 (50%)		
Tumor necrosis [*n* = 132]						
Present	10 (7,6%)	0 (0%)	4 (19%)	6 (66,7%)	< 0,001	0,018
Absent	122 (92,4%)	102 (100%)	17 (81%)	3 (33,3%)		
Angioinvasion [*n* = 119]						
Present	16 (13,4%)	8 (8,5%)	5 (29,4%)	3 (37,5%)	0,028	NS
Absent	103 (86,6%)	86 (91,5%)	12 (70,6%)	5 (62,5%)		
AJCC 8 th pT stage [*n* = 126]						
pT1/T2	54 (42,9%)	50 (51%)	3 (15%)	1 (12,5%)	0,003	NS
pT3/T4	72 (57,1%)	48 (49%)	17 (85%)	7 (87,5%)		
Molecular alterations						
*BRAF V600E* mutated [*n* = 123]	64 (48%)	50 (51,5%)	9 (52,9%)	5 (55,6%)	NS	NS
*NRAS Q61R* mutated [*n* = 91]	1 (1,1%)	1 (1,4%)	0 (0%)	0 (0%)	NS	NS
*TERT* promoter mutated [*n* = 108]	16 (14,8%)	9 (11%)	5 (29,4%)	2 (22,2%)	NS	NS
*− 124G* > *A* mutation	10 (9,3%)	6 (7,3%)	4 (23,5%)	0 (0%)	NS	NS
*− 146G* > *A* mutation	6 (5,6%)	3 (3,7%)	1 (5,9%)	2 (22,2%)	NS	NS
*BRAF V600E* + *TERT* promoter mutation co-occurrence [*n* = 123]	8 (6,5%)	5 (5,2%)	1 (5,9%)	2 (20%)	NS	NS
Follow-up and treatments [*n* = 133]						
Outcome at the end of follow-up [*n* = 137]						
Death due to disease	9 (6,6%)	4 (3,8%)	3 (13,6%)	2 (20%)	NS	NS
Alive with disease	48 (34,8%)	31 (29,5%)	13 (59,1%)	4 (40%)	0,009	NS
Alive without disease	80 (58,4%)	70 (66,7%)	6 (27,3%)	4 (40%)	< 0,001	NS
Additional therapies [*n* = 134]						
Yes	66 (52,8%)	48 (48,5%)	12 (75%)	6 (60%)	0,043	NS
Radioactive iodine therapy	63 (47%)	44 (41,9%)	13 (68,4%)	6 (60%)	NS	NS
External beam radiation therapy	2 (1,6%)	1 (1%)	1 (6,3%)	0 (0%)	NS	NS
Tyrosine kinase inhibitors	2 (1,6%)	0 (0%)	2 (12,5%)	0 (0%)	0,018	NS
Surgery	35 (28%)	22 (22,2%)	7 (43,8%)	6 (60%)	NS	NS
Number of additional RAI administrations [*n* = 134]						
0	71 (53%)	61 (58,1%)	6 (31,6%)	4 (40%)	0,030	NS
1 or more	63 (45,7)	44 (41,9%)	13 (68,4%)	6 (60%)		
Median cumulative dose of RAI in millicuries [mCi] (range) [1 st quartile; 3rd quartile]	141 (30- 34,739,1) [91; 397]	127 (30–1146) [106,5; 507,5]	366 (70–732) [90; 293,5]	280,5 (89–941) [98; 799,5]	0,037	NS

*p* values were obtained from Fisher’s exact tests for categorical variables, and Mann-Whitney U test for continuous variables. When significant, for categorical variables, there are statistical significant differences in the proportion of categories between subgroups (DHGTC vs non-HGDTC or DHGTC vs PDTC), and, for continuous variables, there are statistical significant differences in the distribution of the values in those variables between subgroups (DHGTC vs non-HGDTC or DHGTC vs PDTC)

*PTC*; Papillary Thyroid Cancer, *DHGTC*; Differentiated High Grade Thyroid Carcinoma, *Non-HGDTC*; Non-High Grade Differentiated Thyroid Carcinoma, *PDTC*; Poorly Differentiated Thyroid Carcinoma, *RAI*; Radioactive Iodine, *AJCC 8 th pT stage*; pathological Tumor stage according to the 8 th American Joint Committee on Cancer

# 6 patients presented more than one metastatic localization

### Clinical outcome and statistical analysis

The follow-up period started from the date of primary resection until the end of data collection (June 2013) or when patients were discharged from consultations or died from other causes, and was available for 133 out of 138 patients (median of 78 months; range: 6–320 months). Disease-specific survival (DSS), and persistence of disease at the end of follow-up were chosen to evaluate patient outcome predictors.

DSS was defined as the time from primary resection to death caused by thyroid cancer. Persistence of disease at the end of follow-up was determined by structural disease present at the last visit, confirmed by imaging techniques (CT, MRI, ultrasound and scintigraphy) and biopsies when applicable.

All statistical analyses were conducted using SPSS 29.0 (IBM Corporation, Armonk, NY, USA). Clinicopathological features between subgroups were compared using Fisher’s exact test for categorical variables, and the Mann–Whitney U test for continuous variables. Univariate survival analysis was performed with the log-rank test for categorical variables and with the Cox proportional hazards model for continuous variables. Significant factors from the univariate analysis were further subjected to multivariate analysis with the Cox proportional hazards model. P-values of < 0,05 were considered statistically significant.

## Results

### Clinicopathological and genetic characteristics of the entire cohort

The relevant clinical and pathological features of the entire cohort are summarized in Table 1 and further details are provided in Supplementary Table S1.

The median age was 45 years (range, 13–81 years). There was a female predominance (78,3%). The median tumor size was 2.6 cm (range, 0.5–9 cm).

In terms of diagnosis, 32 cases (23.2%) presented as high-grade follicular cell-derived non-anaplastic thyroid carcinoma, of which 22 (15.9%) were diagnosed as DHGTC and 10 (7.2%) as PDTC.

#### Non-HGDTC vs DHGTC

Among the 106 non-HGDTC cases (76.8%), the most common subtypes were: classical subtype of PTC (33%); infiltrative follicular subtype of PTC (22.6%); invasive encapsulated follicular variant of PTC (8.5%) and oncocytic subtype of PTC (8.5%).

Within the DHGTC cases, the most common subtypes were tall-cell subtype of PTC (TC-PTC) (37.5%); classical subtype of PTC (C-PTC) (22.7%) and infiltrative follicular variant of PTC (IFV-PTC) (13.6%).

The age groups of 18–55 and ≥ 55 displayed significant differences, with the DHGTC group having 8 patients (38.1%) aged 18–55 compared to 77 patients (72.6%) in the non-HGDTC group (*p* < 0.001). Additionally, the DHGTC group had 10 patients (47.6%) aged ≥ 55 compared to 28 patients (26.4%) in the non-HGDTC group (*p* < 0.001).

Distant metastasis was confirmed in 36 out of 138 patients (26.1%), being more frequent in the DHGTC than in non-HGDTC groups: In the DHGTC group, 10 out of 22 patients (45.5%) had distant metastasis compared to 19 out of 106 patients (17.9%) in the non-HGDTC group (*p* = 0.008). Regarding types of distant metastasis, lung metastasis was more frequent in the DHGTC group (36.3%, 8 patients) compared to the non-HGDTC group (11.3%, 12 patients) (*p* = 0.015). Synchronous metastasis was also more common in the DHGTC group (35.3%, 6 patients) than in the non-HGDTC group (14%, 14 patients), (*p* = 0.042). Further details are provided in Supplementary Table S1.

The median tumor size was larger in the DHGTC group (3.5 cm) than in the non-HGDTC group (2.3 cm) (*p* = 0.007). Additionally, tumors > 2 cm were more frequent in the DHGTC group (77.3%, 17 patients) than in the non-HGDTC group (41.5%, 44 patients), (*p* = 0.023).

The analysis of tumor subtypes revealed statistically significant differences between the DHGTC and non-HGDTC groups: TC- PTC was more frequent in the DHGTC group (37.5%, 9 patients vs 7.5%, 8 patients) (*p* < 0.001). By definition, tumor necrosis was only present in the DHGTC group, with 4 out of 21 patients (19%) showing necrosis. Among the 123 cases evaluated, encapsulation was observed as either complete or partial; 80 cases (65%) exhibited infiltrative characteristics and 43 (35%) were capsular invasive. Lymphatic invasion occurred in 31 cases (26.1%), and gross extrathyroidal extension (ETE) was noted in 52 cases (39.4%) but no significant differences were noted between DHGTC and non-HGDTC. Angioinvasion, observed in 16 patients overall, was more frequent in the DHGTC group, 29.4% (*n* = 5) than in the non-HGDTC group, 8.5% (*n* = 8), (*p* = 0.028).

AJCC 8 th pT analysis indicates that 72 out of 126 (57.1%) of the tumors were high stage (T3/T4), 43 out of 137 (31.2%) had pathologically confirmed nodal metastasis (N1a/N1b), and 6 out of 126 (4.8%) had distant metastasis (M1) at diagnosis (Supplementary Table S1).

The analysis of the AJCC 8 th pT stage among 126 patients showed a statistically significant difference between the DHGTC and non-HGDTC groups (*p* = 0.003). In the DHGTC group, 17 out of 20 patients (85%) presented with pT3/T4 stages, compared to 48 out of 98 patients (49%) in the non-HGDTC group (*p* = 0.003).

Almost half of the tumors (48%) had the *BRAF* V600E mutation, and a significant proportion (14,8%) presented *TERT* promoter mutations, whereas *NRAS* mutation was detected in a single case. No differences in the prevalence of the mutations were found between the DHGTC and non-HGDTC groups.

#### DHGTC vs PDTC

When comparing clinicopathological features between DHGTC and PDTC, size, necrosis and mitotic index had come out as statistically significant. The median size for DHGTC was 3.51 cm, while for PDTC was 5.0 cm (*p* = 0.023). Necrosis was present in 4 out of 21 DHGTC patients (19%) compared to 6 out of 9 PDTC patients (66.7%) (*p* = 0.018). The median mitotic index was higher in DHGTC (6 per 2 mm^2^) than in PDTC (4,5 per 2 mm^2^) (*p* = 0.009) and mitotic index ≥ 5 was more frequent in DHGTC (100%) than in PDTC (50%) (*p* < 0,001). Details can be seen in Table 1.

### Outcomes and prognostic factors of the entire cohort

**Fig. 2:**
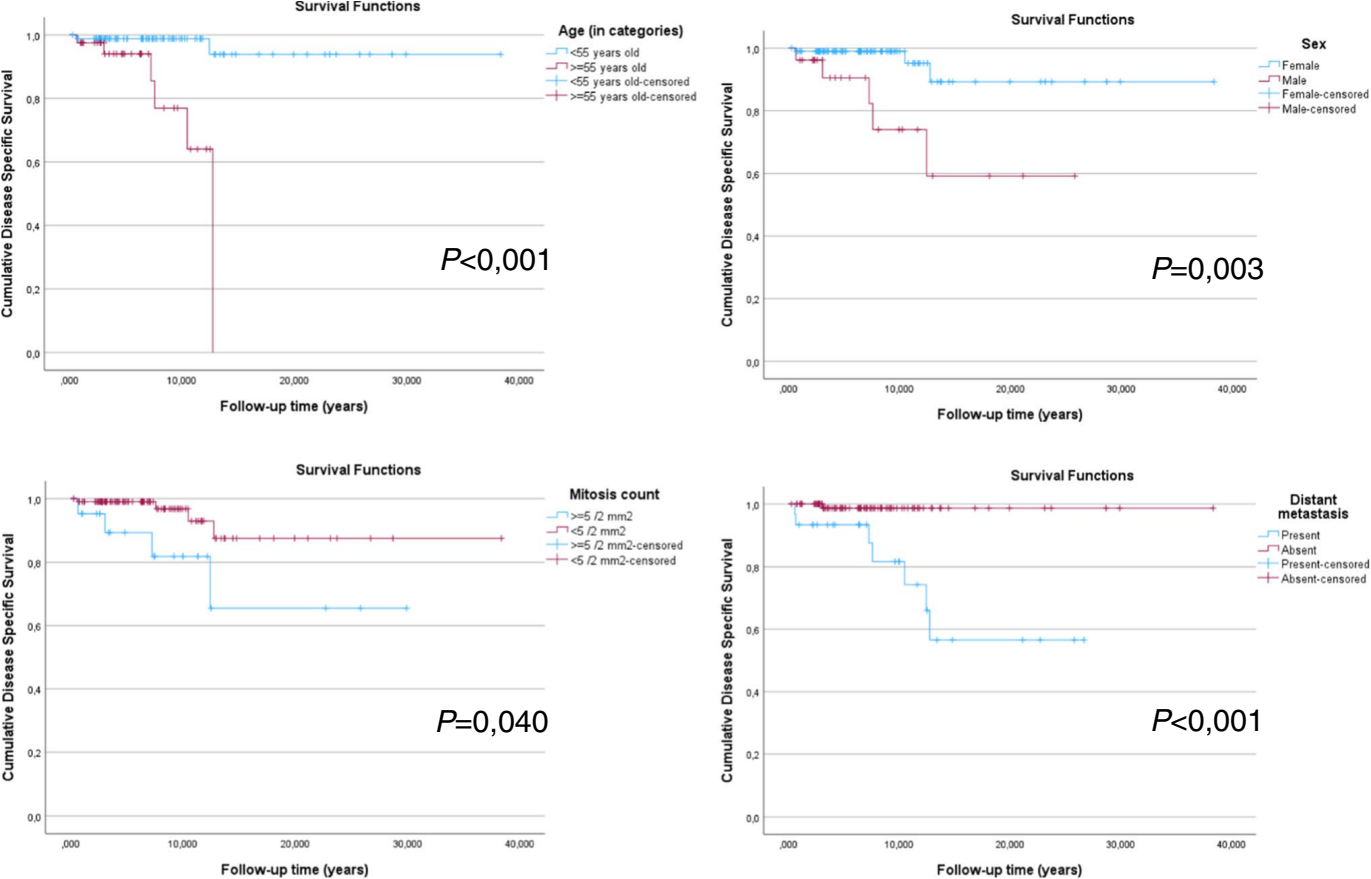
Selected Kaplan–Meier curves for DSS in advanced thyroid cancer

Follow-up data was available for 133 patients, with a median follow-up of 6,54 years (range, 0,28–38,30 years). The overall 3-year; 5-year, 10-year and 20-year DSS rates in the whole series were: 99%; 98%; 96% and 93%; in the patients with DHGTC tumors, these rates were: 95%; 91%; 86%; 86%. Nine (6,6%) patients died due to disease during follow-up, of which 4 (44,4%) were non-HGDTC, 3 DHGTC (33,3%) and 2 (22,2%) PDTC. Univariate survival analysis results on DSS are documented in Table 2, and selected curves of Kaplan–Meier are shown in Fig. 2. Additional Kaplan–Meier curves for DSS can be seen in Supplementary Fig. 1.

**Table 2:** Univariate survival analysis for disease-specific survival (DSS) in advanced thyroid cancer

Clinical parameters	DSS
Age (continuous)	< 0,001
Age (≥ 55 vs < 55 years old)	< 0,001
Sex	0,003
Size (continuous)	0,002
Mitosis count (≥ 5 vs < 5 mitosis per 2 mm^2^)	0,021
Mitosis count (continuous)	0,040
Histotype	0,004
Documented metastasis	0,019
Lymph node metastasis	NS
Distant metastasis	< 0,001
Bone metastasis	< 0,001
Lymphatic invasion	NS
Synchronous metastasis	< 0,001
AJCC 8 th pT stage (pT3/T4 vs pT1/T2)	0,047
AJCC 8 th pM stage (pM1 vs pM0/Mx)	0,041

Prognostic factors identified on univariate analysis that were found to be statistically significant for DSS were age (continuous and categorical) (*p* < 0,001), sex (*p* = 0,003), size (continuous) (*p* = 0,002), the mitotic index category (*p* = 0.021) and continuous (*p* = 0,04), histotype (*p* = 0,004), documented metastasis (*p* = 0,019), distant metastasis (*p* < 0,001), bone metastasis (*p* < 0,001), synchronous metastasis (*p* < 0,001), AJCC 8 th pT stage (pT3/T4 vs pT1/T2) (*p* = 0,047), AJCC 8 th pM stage (*p* = 0,041), persistence of disease after one year (*p* = 0,047) and cumulative dose of RAI (*p* = 0,026). Further details can be seen in Table 2.

The results of multivariate survival analysis with the Cox proportional hazards model are shown in Table 3. Independent prognostic factors for worse DSS were age (≥ 55 vs < 55 years old) [*p* = 0,005, HR = 19,625, 95% CI (2,479–155,372)] and sex (male vs female) [*p* = 0,029, HR = 7,441, 95% CI (1,231–44,965)].

**Table 3:** Multivariate survival analysis for DSS using Cox proportional hazards model in advanced thyroid cancer*

DSS		
Age (≥ 55 vs < 55 years old)	**0,005**	19,625 (2,479–155,372)
Sex (male vs female)	**0,029**	7,441 (1,231–44,965)
Size (continuous)	0,132	1,034 (1,007–1,063)
Lymph node metastasis	0,161	3,226 (0,237–7,658)
Distant metastasis	0,056	2,710 (0,972–7,874)
Mitosis count	0,899	1,020 (0,742–1,410)
AJCC 8 th pT stage (pT3/T4 vs pT1/T2)	0,702	1,263 (0,330–3,759)

*Redundant variables were excluded from the multivariate analysis to prevent the occurrence of incorrect interactions. Bold figures indicate significant differences

### Outcomes and prognostic factors of patients with DHGTC

No prognostic factors were found on survival analysis for DSS in the DHGTC subgroup.

## Discussion

The 5 th edition of WHO’s Classification of Thyroid Neoplasms introduced a new entity -DHGTC- to incorporate prognostic value into the histological classification. To date, the literature on this topic is sparse, with only a few studies with varying methodologies addressing the issue. In order to apply that concept within a cohort of thyroid carcinomas with advanced disease characteristics (based on the 2022 AHNS guidelines) [18], we reclassified, reanalysed and compared the pathological, molecular, and clinical characteristics of DHGTC against both non-HGDTC and PDTC, together with long-term follow-up data.

In our series of advanced thyroid carcinomas, the prevalence of DHGTC is higher than what is described in studies without prior clinical selection (17,2% vs 7,2%) [23]. We observed significant differences in age distribution between DHGTC and non-HGDTC groups, with a higher proportion of patients aged ≥ 55 years in the DHGTC group (*p* < 0.001), as previously advanced by other authors [10, 13–15]. In our series, tumor size was also consistently larger in the DHGTC than in non-HGDTC group as reported across the reviewed studies [10, 13–15].

The most common subtypes of PTC in the present series were classical and infiltrative follicular PTC. However, within the DHGTC group, the most prevalent subtype was TC-PTC, known for its aggressiveness and associated poorer prognosis. This aligns with findings from previous studies, where TC-PTC is also predominant in the DHGTC group [10, 12, 14, 15].

Resulting from the definition of DHGTC, tumor necrosis was observed in 19% of the DHGTC and absent in non-HGDTC (*p* < 0.001), and increased mitotic activity was found in DHGTC (median of 6 per 2 mm^2^ vs. 1 per 2 mm^2^ in non-HGDTC, *p* < 0.001). Furthermore, the occurrence of angioinvasion was significantly more frequent in DHGTC than in non-HGDTC. These findings were parallel with those of Jeong et al. [15], who analyzed 106 non-HGDTC and 32 DHGTC cases, identifying statistically significant differences in advanced pathological features between DHGTC over non-HGDTC, and with the pioneering study by Xu et al. [12] which also documented higher (T3/T4) AJCC 8 th pT stage, microscopic ETE, and vascular invasion in DHGTC. Taking together our results and those from previous works, the new DHGTC entity robustly signals more aggressive carcinomas, and notably, even within advanced thyroid carcinomas, as is the case of the present series.

Although our primary goal was to highlight differences between DHGTC and non-HGDTC in a dataset enriched in advanced thyroid carcinoma, since our series also includes 10 cases of PDTC, it became pertinent to examine putative prognostic differences between DHGTC and PDTC as two subgroups of high-grade follicular cell-derived non-anaplastic thyroid carcinoma. In a previous work, Jeong et al. found in their cohort of 77 PDTC cases that, when compared to DTCs without high-grade features, PDTCs had higher rates of vascular invasion, lymphatic invasion, and gross ETE [15]. Now, when we compare DHGTC with PDTC, our study revealed that PDTCs showed significantly larger tumor sizes (*p* = 0.023) and higher frequency of tumor necrosis (*p* = 0.018) in comparison with DHGTC, whereas DHGTC has significantly higher mitotic index, although this later difference is probably a consequence of the DHGTC definition. In the study by Wong et al. [10], where the authors compared 15 PTCs with high-grade features (PTC-HGF) and 47 PDTCs cases, the authors report higher rates of pT4 disease and lymph node metastasis in PTC-HGF vs. PDTC, indicating that PTC-HGF can present more aggressive pathological profile than PDTC. Taken together, our and Wong’s study [10], highlight critical differences in terms of aggressiveness, based on pathological characteristics. Of note, the term DHGTC is already included in guidelines, as a pre-or intra-op indication for total thyroidectomy and treatment with adjuvant RAI in tumors diagnosed as DTC in fine needle aspiration [24].

Our results and the aforementioned studies confirm the need of grading well-differentiated thyroid carcinomas, regardless of the subtype [10, 12–15]. Aligning the mitosis and necrosis-based grading system seems effective in segregating cases with advanced pathological characteristics. However, when we compare DHGTC vs. PDTC, this profile of segregation becomes less clear [25]. Overall, these studies highlight the distinct pathological profiles and aggressive behaviour of DHGTC and PDTC, emphasizing the need for accurate classification and management of these high-grade thyroid carcinomas. Resta et al. [13], while acknowledging limitations in their study of 32 DHGTCs (without any PDTC cases), namely insufficient long-term follow-up, noted that, although DHGTCs resemble well-differentiated variants, they tend to present more aggressively, often with metastases, though not as aggressively as PDTCs.

Jeong et al. [15] observed *TERT* promoter mutations in 42.9% of DHGTC cases, significantly more frequent than the 1.4% in DTC cases, with no significant differences in *BRAF* mutations between the groups. *TERT* promoter mutations were also more commonly seen in PDTCs than in DTCs without high-grade features [15]. Additionally, the same study indicated a trend of higher *BRAF* V600E mutations in DHGTCs compared to PDTCs [15]. This aligns with the research by Wong et al. and Xu et al. [10, 12], who also reported a higher occurrence of *BRAF* V600E and fewer *RAS* mutations in DHGTCs, largely due to the prevalent PTC histology within this group. Resta et al. [13] highlighted *TERT* promoter mutations as indicators of aggressive behaviour, but their analysis was constrained by only eight cases with molecular profile and the absence of PDTC in the series. Wong et al. [10] suggest that *BRAF* V600E mutations are specifically linked to DHGTCs. Our study, (encompassing 138 cases, including 22 DHGTCs and 10 PDTCs) includes mutational analysis for *BRAF* V600E, *NRAS* Q61R, and *TERT* promoter in a majority of the cases. We were able to perform a detailed comparison, yet we did not find significant differences in mutation frequencies in the comparisons between DHGTC vs non-HGDTC or DHGTC vs PDTC. Similar frequencies in *BRAF* and *RAS* mutations were found in the 3 groups of tumors and, although DHGTC and PDTC present higher frequencies of *TERT* promoter mutations, no significant differences were found. This similarity in genetic alterations can be related with the fact that we are leading with a series of advanced tumors (recurrent and/or metastatic) where the molecular characteristics might be more closely aligned, highlighting the need for further comprehensive studies.

When considering clinical endpoints (DSS), the pattern observed between comparison groups remains consistent with the findings of clinicopathologic features. Revisiting the follow-up duration across studies, our study represents the longest duration reported, with a median of 78 months (range: 6–320 months). Although the specific metrics for clinical endpoints vary from study to study, DHGTC cases consistently show worse clinical outcomes compared to non-HGDTC cases, including lower survival rates and more cases with persistence of disease at the end of follow-up [10, 14, 15]. In our study, we noted lower 3-year; 5-year, 10-year and 20-year DSS rates for the DHGTC group and identified adverse prognostic clinicopathological factors affecting DSS in the whole population. Xu et al. [12] also had a long-term follow-up with a median of 56 months and showed that DSS, DMFS, and LRRFS were lower for the DHGTC group compared to the non-HGDTC group. Jeong et al. [15] documented that RFS was significantly shorter in patients with DHGTCs and PDTC compared to patients with DTC without HG features, while the RFS duration did not differ significantly between patients with DHGTCs and PDTCs. In Ghossein et al.’s most recent study comparing Papillary thyroid carcinoma tall cell subtype (PTC-TC) and high-grade differentiated thyroid carcinoma tall cell phenotype (HGDTC-TC) [16], they found HGDTC-TC was associated with a significantly decreased DSS, LRDFS and distant metastasis-free survival, proving that, even within PTC with known aggressive features, DHGTC classification distinguishes worse clinical outcomes.

Univariate analysis of the whole series revealed that the significant parameters for DSS, were age (continuous and categoric), sex, size, mitotic count, histotype, documented metastasis, distant metastasis, bone metastasis, synchronous metastasis, AJCC 8 th pT stage (pT3/T4 vs pT1/T2 and AJCC 8 th pM stage. In subsequent multivariate analysis, only age and sex were significant independent prognostic factors for worse DSS.

In the univariate survival analysis restricted to DHGTC, no factors were independently associated with worse DSS, likely due to the limited number of deceased patients (*n* = 9), since advanced thyroid cancer rarely leads to death but often results in persistent disease. In addition, low numbers in subgroup analyses and interactions between factors often lead to a lack of significance in multivariate analysis.

On a side note, in our dataset the number of cases which developed radioactive iodine-refractory (RAIR) thyroid cancer (*n* = 12; Supplementary Table S2) was significant, given the estimated incidence of 4–5 cases/year/million people [4, 26]. The most frequent diagnosis in RAIR thyroid cancer was PDTC, consistent with its poor prognosis. All RAIR cases presented with distant metastasis and represented some of the most aggressive forms in our series, including cases with brain and kidney metastases. These cases often exhibited adverse clinicopathological features such as capsular invasion, lymphatic invasion, angioinvasion, minimal ETE, infiltrative and invasive capsule status, high AJCC pT stage, *BRAF* V600E mutations, *TERT* promoter mutations, lymph node metastasis, and synchronous metastasis [26].

Our study has some limitations: firstly, it lacks advantages of a prospective study due to its retrospective design and it involves reclassification of lesions. It was not initially designed to compare DHGTC with PDTC given the well-established profile of PDTC in the literature, potentially limiting the comprehensiveness of findings concerning the full spectrum of DHGTCs. Additionally, the small number of cases in some subgroups may reduce the statistical power necessary to detect significant differences in certain pathological and molecular features.

In conclusion, this study stands out for its comprehensive clinical and pathological analysis, extensive mutation profiling, and the inclusion of longest-term follow-up data available in the literature focusing on this new entity “DHGTC”, and thus, provides valuable insights into the behaviour and management of advanced thyroid carcinomas. Interestingly, the study delineates the variability in aggressiveness and prognosis within high-grade thyroid carcinomas as pointed by the recent and comprehensive review article of Coca-Pelaz et al. [25]. Furthermore, the alignment with the latest WHO classification’s criteria ensures the relevance and applicability of our findings to current clinical practice. Our results support the usefulness of subgrouping the tumors presenting DHGTC features under high-grade follicular cell-derived non-anaplastic thyroid carcinomas, as proposed in the 5 th WHO classification, since they more frequently display aggressive features and poor outcomes.

## Supplementary information

Below is the link to the electronic supplementary material.

ESM 1 (DOCX 272 KB)

## Data Availability

Dataset were deposited in the files of “Cancer Signaling and Metabolism” research group of i3 s. Access to the dataset used might be possible by contacting with corresponding author.

## Declarations

The clinicopathological data was collected from clinical records, with the approval of the Ethic Committee of the Faculty of Medicine of the University of Coimbra (n° 1309) and all the procedures described in this study were done in accordance with national ethical standards (Law n° 12/2005) and Helsinki declaration.
