# Supplementary material for: Comparative analysis of follicular cell- derived thyroid carcinoma: assessing the impact of high-grade features in an advanced disease cohort
Source: Virchows Arch. 2025 May 2;486(6):1305–15. doi: 10.1007/s00428-025-04109-2 (PMC12214025; doi:10.1007/s00428-025-04109-2)
Supplement: Supplementary file 1 — (DOCX 272 KB) [file 428_2025_4109_MOESM1_ESM.docx]

Supplementary materials:

*Table S1.* Clinicopathologic characteristics of 138 patients with primarily resected advanced thyroid cancer.

|  | **All [n=138]** | **DHGTC [n=22]** | **Non-HGDTC [n=106]** | **PDTC**  **[n=10]** | ***P* value (DHGTC vs non-HG-DTC)** | ***P* value**  **DHGTC vs PDTC)** |
| --- | --- | --- | --- | --- | --- | --- |
| **Clinicopathological parameters** | | | | | | |
| **Age (years) [n=137]** | | | | | | |
| Median (range) [1^st^ quartile; 3^rd^ quartile] | 45 (13-81) [34;57,5] | 44,5 (17-74) [34;56] | 48 (13-81) [31; 71,5] | 53 (22-69) [42; 65,5] | NS | NS |
| ≤18 | 4 (3,1%) | 1 (0,9%) | 3 (13,6%) | 0 (0%) | 0,016 |  |
| 18-55 | 91 (65,9%) | 77 (72,6%) | 8 (38,1%) | 6 (60%) | <0,001 | NS |
| ≥55 | 42 (30,4%) | 28 (26,4%) | 10 (47,6%) | 4 (40%) | <0,001 |  |
| **Sex** | | | | | | |
| Female | 108 (78,3%) | 16 (72,7%) | 86 (81,1%) | 6 (60%) | NS | NS |
| Male | 30 (21,7%) | 6 (27,3%) | 20 (18,9%) | 4 (40%) |  |  |
| **Documented metastasis** | 85 (61,6%) | 16 (72,7%) | 62 (58,5%) | 7 (70%) | NS | NS |
| **Distant metastasis [n=36]** | 36 (26,1%) | 19 (17,9%) | 10 (45,5%) | 7 (70%) | 0,008 | NS |
| **Types of distant metastasis [n=36] #** | | | | | | |
| Bone | 13 (9,4%) | 8 (7,5%) | 3 (13,6%) | 2 (20%) | NS | NS |
| Lung | 26 (18,8%) | 12 (11,3%) | 8 (36,3%) | 6 (20%) | 0,015 |  |
| Brain | 2 (1,4%) | 0 (0%) | 1 (4,5%) | 1 (10%) | NS |  |
| Kidney | 1 (0,7%) | 0 (0%) | 1 (4,5%) | 0 (0%) | NS |  |
| **Lymph node metastasis [n=135]** | 67 (49,6%) | 12 (60%) | 52 (49,5%) | 3 (30%) | NS | NS |
| **Synchronous metastasis [n=127]** | 27 (21,3%) | 6 (35,3%) | 14 (14%) | 7 (70%) | 0,042 | NS |
| **Tumor size (cms) [n=125]** | | | | | | |
| Median (range) [1^st^ quartile; 3^rd^ quartile] | 2,65 (0,5-9) | 2,3 (0,5-9) [1,5; 2,7] | 3,51 (0,8-8) [2,0; 4,8] | 5,0 (2,5-8)  [2,0; 4,4] | 0,007 | 0,023 |
| ≤2 cm | 67 (56,3%) | 5 (22,7%) | 62 (58,5%) | 0 (0%) | 0,023 | NS |
| >2 cm | 61 (47,7%) | 17 (77,3%) | 44 (41,5%) | 8 (100%) |  |  |
| **Main diagnosis** | | | | | | |
| Classical subtype of PTC | 40 (29%) | 5 (22,7%) | 35 (33%) | NA | NS | NA |
| Hobnail subtype of PTC | 6 (4,3%) | 1 (4,5%) | 5 (4,7%) | NA | NS | NA |
| Invasive encapsulated follicular subtype of PTC | 9 (6,5%) | 0 (0%) | 9 (8,5%) | NA | NS | NA |
| Infiltrative follicular subtype of PTC | 27 (19,6%) | 3 (13,6%) | 24 (22,6%) | NA | NS | NA |
| Oncocytic subtype of PTC | 9 (6,5%) | 0 (0%) | 9 (8,5%) | NA | NS | NA |
| Solid subtype of PTC | 6 (4,3%) | 2 (9,1%) | 4 (3,8%) | NA | NS | NA |
| Tall-cell subtype of PTC | 17 (12,3) | 9 (37,5%) | 8 (7,5%) | NA | <0,001 | NA |
| Warthin-like subtype of PTC | 7 (5,1%) | 0 (0%) | 7 (6,6%) | NA | NS | NA |
| Oncocytic carcinoma of the thyroid | 2 (1,4%) | 1 (4,5%) | 1 (0,9%) | NA | NS | NA |
| Follicular thyroid carcinoma | 5 (3,6%) | 1 (4,5%) | 4 (3,8%) | NA | NS | NA |
| Poorly differentiated thyroid carcinoma | 10 (7,2%) | NA | NA | 10 (100%) | NA | NA |
| **Mitotic index (per 2 mm^2^)** | | | | | | |
| Median (range) [1^st^ quartile; 3^rd^ quartile] | 2 (0-8) [1;4] | 1 (0-4) [1;2] | 6 (5-8)] [5; 6,25] | 4,5 (3-8) [3,75; 5,25] | <0,001 | 0,009 |
| <5 | 111 (80,4%) | 0 (0%) | 106 (100%) | 5 (50%) | <0,001 | <0,001 |
| ≥5 | 27 (19,6%) | 22 (100%) | 0 (0%) | 5 (50%) |  |  |
| **Tumor necrosis [n=132]** | | | | | | |
| Present | 10 (7,6%) | 4 (19%) | 0 (0%) | 6 (66,7%) | <0,001 | 0,018 |
| Absent | 122 (92,4%) | 17 (81%) | 102 (100%) | 3 (33,3%) |  |  |
| **Encapsulation [n=123]** | | | | | | |
| Encapsulated | 91 (74%) | 11 (64,7%) | 73 (75,3%) | 7 (77,8%) | NS | NS |
| Partially encapsulated | 32 (26%) | 6 (35,3%) | 24 (24,7%) | 2 (22,2%) |  |  |
| **Capsular invasion [n=114]** | | | | | | |
| Present | 108 (94,7%) | 16 (100%) | 83 (93,3%) | 9 (100%) | NS | NS |
| Absent | 6 (5,3%) | 0 (0%) | 6 (6,7%) | 0 (0%) |  |  |
| **Lymphatic invasion [n=119]** | | | | | | |
| Present | 31 (26,1%) | 4 (23,5%) | 23 (24,5%) | 4 (50%) | NS | NS |
| Absent | 88 (73,9%) | 13 (76,5%) | 71 (75,5%) | 4 (50%) |  |  |
| **Angioinvasion [n=119]** | | | | | | |
| Present | 16 (13,4%) | 5 (29,4%) | 8 (8,5%) | 3 (37,5%) | 0,028 | NS |
| Absent | 103 (86,6%) | 12 (70,6%) | 86 (91,5%) | 5 (62,5%) |  |  |
| **Gross extrathyroidal extension [n=132]** | | | | | | |
| Present | 52 (39,4%) | 8 (38,1%) | 42 (41,2%) | 2 (22,2%) | NS | NS |
| Fibroadipose tissue | 48 (36,3%) | 7 (31,8%) | 39 (36,8%) | 2 (20%) | NS | NS |
| Skeletal muscle | 7 (5,3%) | 1 (5%) | 6 (5,7%) | 0 (0%) | NS | NS |
| Other organs | 1 (0,8%) | 0 (0%) | 1 (0,9%) | 0 (0%) | NS | NS |
| **Capsule status [n=123]** | | | | | | |
| Infiltrative | 80 (65%) | 9 (52,9%) | 69 (71,1%) | 2 (22,2%) | NS | NS |
| Invasive | 43 (35%) | 8 (47,1%) | 28 (28,9%) | 7 (77,8%) |  |  |
| **AJCC 8^th^ pT stage [n=126]** | | | | | | |
| pT1/T2 | 54 (42,9%) | 3 (15%) | 50 (51%) | 1 (12,5%) | 0,003 | NS |
| pT3/T4 | 72 (57,1%) | 17 (85%) | 48 (49%) | 7 (87,5%) |  |  |
| **AJCC 8^th^ pN stage** | | | | | | |
| pN0/Nx | 95 (73%) | 16 (72,7%) | 77 (72,6%) | 8 (80%) | NS | NS |
| pN1a/N1b | 43 (31,2%) | 6 (27,3%) | 37 (27,4%) | 2 (20%) |  |  |
| **AJCC 8^th^ pM stage** | | | | | | |
| pM0/Mx | 132 (95,7%) | 21 (95,5%) | 103 (97,2% | 8 (80%) | NS | NS |
| pM1 | 6 (4,3%) | 1 (4,5%) | 3 (2,8%) | 2 (20%) |  |  |
| **Molecular alterations** | | | | | | |
| *BRAF V600E* mutated [n=123] | 64 (48%) | 9 (52,9%) | 50 (51,5%) | 5 (55,6%) | NS | NS |
| *NRAS Q61R* mutated [n=91] | 1 (1,1%) | 0 (0%) | 1 (1,4%) | 0 (0%) | NS | NS |
| *TERT* promoter mutated [n=108] | 16 (14,8%) | 5 (29,4%) | 9 (11%) | 2 (22,2%) | NS | NS |
| *-124G>A* mutation | 10 (9,3%) | 4 (23,5%) | 6 (7,3%) | 0 (0%) | NS | NS |
| *-146G>A* mutation | 6 (5,6%) | 1 (5,9%) | 3 (3,7%) | 2 (22,2%) | NS | NS |
| **Follow-up and treatments [n=133]** | | | | | | |
| Median follow-up time in years (range) [n=117] | 6,54 (0,28-38,3) | 4,34 (0,67-29,9) | 6,57 (0,28-38,3) | 8,78 (3,95-21,13) | NS | NS |
| Median time until the first recurrence in months (range) | 17,32 (0,07-312,8) | 26,40 (0,83-312,8) | 15,80 (0,07-93,21) | 47,71 (8,17-104,9) | NS | NS |
| Outcome at the end of follow-up [n=137] | | | | | | |
| Death due to disease | 9 (6,6%) | 3 (13,6%) | 4 (3,8%) | 2 (20%) | NS | NS |
| Alive with disease | 48 (34,8%) | 13 (59,1%) | 31 (29,5%) | 4 (40%) | 0,009 | NS |
| Alive without disease | 80 (58,4%) | 6 (27,3%) | 70 (66,7%) | 4 (40%) | <0,001 | NS |
| Development of radioactive iodine refractoriness (RAIR) | 12 (8,7%) | 3 (13,6%) | 5 (4,7%) | 4 (40%) | NS | NS |
| Number of recurrences [n=117] | | | | | | |
| 1 | 62 (53%) | 11 (64,7%) | 45 (50%) | 6 (60%) | NS | NS |
| 2 | 55 (47%) | 6 (35,3%) | 45 (50%) | 4 (40%) |  |  |
| Additional therapies [n=134] | | | | | | |
| Yes | 66 (52,8%) | 12 (75%) | 48 (48,5%) | 6 (60%) | 0,043 | NS |
| Radioactive iodine therapy (RAI) | 63 (47%) | 13 (68,4%) | 44 (41,9%) | 6 (60%) | NS | NS |
| Radiation therapy | 2 (1,6%) | 1 (6,3%) | 1 (1%) | 0 (0%) | NS | NS |
| Tyrosine kinase inhibitors | 2 (1,6%) | 2 (12,5%) | 0 (0%) | 0 (0%) | 0,018 | NS |
| Surgery | 35 (28%) | 7 (43,8%) | 22 (22,2%) | 6 (60%) | NS | NS |
| Number of additional RAI administrations [n=134] | | | | | | |
| 0 | 71 (53%) | 6 (31,6%) | 61 (58,1%) | 4 (40%) | 0,030 | NS |
| 1 or more | 63 (45,7) | 13 (68,4%) | 44 (41,9%) | 6 (60%) |  |  |
| Median cumulative dose of RAI in millicuries [mCi] (range) [1^st^ quartile; 3^rd^ quartile] | 141 (30-  34739,1) [91; 397] | 127 (30-1146) [106,5; 507,5] | 366 (70-732) [90; 293,5] | 280,5 (89-941) [98; 799,5] | 0,037 | NS |

*p values were obtained from Fisher’s exact tests for categorical variables, and Mann-Whitney U test for continuous variables. When significant, for categorical variables, there are statistical significant differences in the proportion of categories between subgroups (DHGTC vs non-HGDTC or* *DHGTC vs PDTC), and, for continuous variables, there are statistical significant differences in the distribution of the values in those variables between subgroups (DHGTC vs non-HGDTC or* *DHGTC vs PDTC).*

*Abbreviations: PTC: Papillary Thyroid Cancer; DHGTC: Differentiated High Grade Thyroid Carcinoma; Non-HGDTC: Non-High Grade Differentiated Thyroid Carcinoma; PDTC: Poorly Differentiated Thyroid Carcinoma; RAI: Radioactive Iodine; AJCC 8th pT stage: pathological Tumor stage according to the 8^th^ American Joint Committee on Cancer; AJCC 8th pN stage: pathological Lymph node stage according to the 8^th^ American Joint Committee on Cancer; AJCC 8th pM stage: pathological Metastasis stage according to the 8^th^ American Joint Committee on Cancer*

*# 6 patients presented more than one metastatic localization*

**Table S2.** Clinicopathologic characteristics of 12 patients with primarily resected RAIR thyroid cancer

|  | **All [n=12]** | **DHGTC [n=3]** | **Non-HGDTC [n=5]** | **PDTC [n=4]** |
| --- | --- | --- | --- | --- |
| **Clinicopathological parameters** | | | |  |
| **Age (years)** | | | |  |
| Median (range) | 47,5 (15-74) | 44 (15-45) | 63 (44-74) | 52 (30-69) |
| ≤18 | 1 (8,3%) | 1 (33,3%) | 0 (0%) | 0 (0%) |
| 18-55 | 7 (58,3%) | 2 (66,7%) | 2 (40% | 3 (75%) |
| ≥55 | 4 (33,3%) | 0 (0%) | 3 (60%) | 1 (25%) |
| **Sex** | | | |  |
| Female | 10 (83,%) | 3 (100%) | 4 (80%) | 3 (75%) |
| Male | 2 (16,7%) | 0 (0%) | 1 (20%) | 1 (25%) |
| **Documented metastasis** | 12 (100%) | 3 (100%) | 5 (100%) | 4 (100%) |
| **Distant metastasis** | 12 (100%) | 3 (100%) | 5 (100%) | 4 (100%) |
| **Types of distant metastasis #** | | | |  |
| Bone | 4 (33,3%) | 0 (0%) | 3 (60%) | 1 (25%) |
| Lung | 9 (75%) | 1 (33,3%) | 1 (20%) | 4 (100%) |
| Brain | 1 (8,3%) | 1 (33,3%) | 0 (0%) | 0 (0%) |
| Kidney | 1 (8,3%) | 1 (33,3%) | 0 (0%) | 0 (0%) |
| **Lymph node metastasis** | 7 (58,3%) | 1 (33,3%) | 4 (80%) | 2 (50%) |
| **Synchronous metastasis** | 10 (83,3%) | 3 (100%) | 3 (60%) | 4 (100%) |
| **Tumor size (cms) [n=9]** | | | |  |
| Median (range) | 2,50 (1,50-8,00) | 3,25 (2,50-40) | 2,00 (1,50-2,80) | 6,00 (4,00-8,00) |
| ≤2 cm | 3 (25%) | 0 (0%) | 3 (60%) | 0 (0%) |
| >2 cm | 6 (75%) | 3 (100%) | 2 (40%) | 2 (100%) |
| **Main diagnosis** | | | |  |
| Classical subtype of PTC | 2 (16,7%) | 1 (33,3%) | 1 (20%) | NA |
| Infiltrative follicular subtype of PTC | 2 (16,7%) | 1 (33,3%) | 1 (20%) | NA |
| Oncocytic subtype of PTC | 1 (8,3%) | 0 (0%) | 1 (20%) | NA |
| Warthin-like subtype of PTC | 1 (8,3%) | 0 (0%) | 1 (20%) | NA |
| Oncocytic carcinoma of the thyroid | 1 (8,3%) | 1 (33,3%) | 0 (0%) | NA |
| Follicular thyroid carcinoma | 1 (8,3%) | 0 (0%) | 1 (20%) | NA |
| Poorly differentiated thyroid carcinoma | 4 (33,3%) | NA | NA | 4 (100%) |
| **Mitotic index (per 2 mm^2^)** | | | |  |
| Median (range) | 4,5 (1-8) | 6 (5-6) | 2 (1-3) | 5 (4-8) |
| <5 | 6 (50%) | 0 (0%) | 5 (100%) | 1 (25%) |
| ≥5 | 6 (50%) | 3 (100%) | 0 (0%) | 3 (75%) |
| **Tumor necrosis [n=11]** | | | |  |
| Present | 2 (16,7%) | 0 (0%) | 0 (0%) | 2 (66,7%) |
| Absent | 9 (81,8%) | 3 (100%) | 5 (100%) | 1 (33,3%) |
| **Encapsulation [n=11]** | | | |  |
| Encapsulated | 9 (81,8%) | 3 (100%) | 4 (80%) | 2 (66,7%) |
| Partially encapsulated | 2 (18,2%) | 0 (0%) | 1 (20%) | 1 (33,3%) |
| **Capsular invasion [n=11]** | | | |  |
| Present | 11 (100%) | 3 (100%) | 5 (100%) | 3 (100%) |
| **Lymphatic invasion [n=10]** | | | |  |
| Present | 6 (60%) | 1 (33,3%) | 1 (20%) | 2 (100% |
| Absent | 4 (40%) | 2 (66,7%) | 4 (80%) | 0 (0%) |
| **Angioinvasion [n=10]** |  |  |  |  |
| Present | 5 (50%) | 2 (66,7%) | 2 (40%) | 1 (50%) |
| Absent | 5 (50%) | 1 (33,3%) | 3 (60%) | 1 (50%) |
| **Gross extrathyroidal extension [n=11]** | | | |  |
| Present | 7 (63,6%) | 2 (66,7%) | 3 (60%) | 2 (66,7%) |
| Fibroadipose tissue | 6 (54,5%) | 2 (66,7%) | 3 (60%) | 2 (66,7%) |
| Skeletal muscle | 1 (9,1%) | 1 (33,3%) | 1 (20%) | 0 (0%) |
| Other organs | 1 (9,1%) | NA | NA | 0 (0%) |
| **Capsule status [n=11]** | | | |  |
| Infiltrative | 8 (72,7%) | 2 (66,7%) | 4 (80%) | 2 (66,7%) |
| Invasive | 3 (27,3%) | 1 (33,3%) | 1 (20%) | 1 (33,3%) |
| **AJCC 8^th^ pT stage [n=11]** | | | |  |
| pT1/T2 | 3 (27,3%) | 0 (0%) | 2 (40%) | 1 (33,3%) |
| pT3/T4 | 8 (72,7%) | 3 (100%) | 3 (60%) | 2 (66,7%) |
| **AJCC 8^th^ pN stage** | | | |  |
| pN0/Nx | 8 (66,7%) | 3 (100%) | 3 (60%) | 2 (50%) |
| pN1a/N1b | 4 (33,3%) | 0 (0%) | 2 (40%) | 2 (50%) |
| **AJCC 8^th^ pM stage** |  |  |  |  |
| pM0/Mx | 9 (75%) | 3 (100%) | 3 (60%) | 3 (75%) |
| pM1 | 3 (25%) | 0 (0%) | 2 (40%) | 1 (25%) |
| **Molecular alterations** | | | |  |
| *BRAF V600E* mutated [n=11] | 6 (54,5%) | 0 (0%) | 3 (60%) | 2 (50%) |
| *NRAS Q61R* mutated [n=11] | 0 (0%) | 0 (0%) | 0 (0%) | 0 (0%) |
| *TERT* promoter mutated | 4 (33,3%) | 2 (66,7%) | 1 (20%) | 1 (25%) |
| *-124G>A* mutation | 2 (16,7%) | 1 (33,3%) | 1 (20%) | 0 (0%) |
| *-146G>A* mutation | 2 (16,7%) | 1 (33,3%) | 0 (0%) | 1 (25%) |
| **Follow-up and treatments** | | | |  |
| Median follow-up time in years (range) | 10 (2,60-22,7) | 3,53 (2,60-22,7) | 9,97 (6,35-12,76) | 11,27 (7,26-14,80) |
| Median time until the first recurrence in months (range) | 10,2 (0,83-88,9) | 9,3 (0,83-43,5) | 9,10 (7,37-21-60) | 31,00 (81,17-88,90) |
| Outcome at the end of follow-up | | | |  |
| Death due to disease | 3 (25%) | 0 (0%) | 2 (40%) | 1 (25%) |
| Alive with disease | 8 (66,7%) | 3 (100%) | 2 (40%) | 3 (75%) |
| Alive without disease | 1 (8,3%) | 0 (0%) | 1 (20%) | 0 (0%) |
| Number of recurrences [n=11] | | | |  |
| 1 | 11 (100%) | 2 (100%) | 5 (100%) | 4 (100%) |
| Additional therapies | | | |  |
| None | 0 (0%) | 0 (0%) | 0 (0%) | 0 (0%) |
| Radioactive iodine therapy (RAI) | 12 (100%) | 3 (100%) | 5 (100%) | 4 (100%) |
| Radiation therapy | 0 (0%) | 0 (0%) | 0 (0%) | 0 (0%) |
| Tyrosine kinase inhibitors | 2 (16,7%) | 2 (66,7%) | 0 (0%) | 0 (0%) |
| Surgery | 11 (91,7%) | 3 (100%) | 4 (80%) | 4 (100%) |
| Number of additional RAI administrations | | | |  |
| 0 | 0 (0%) | 0 (0%) | 0 (0%) | 0 (0 %) |
| 1 or more | 12 (100%) | 3 (100%) | 5 (100%) | 4 (100%) |
| Median cumulative dose of RAI in millicuries [mCi] (range) | 729 (241-1094) | 501 (498-659) | 812 (241-1094) | 801 (381-941) |

*Abbreviations: PTC: Papillary Thyroid Cancer; DHGTC: Differentiated High Grade Thyroid Carcinoma; Non-HGDTC: Non-High Grade Differentiated Thyroid Carcinoma; PDTC: Poorly Differentiated Thyroid Carcinoma; RAI: Radioactive Iodine; AJCC 8th pT stage: pathological Tumor stage according to the 8^th^ American Joint Committee on Cancer; AJCC 8th pN stage: pathological Lymph node stage according to the 8^th^ American Joint Committee on Cancer; AJCC 8th pM stage: pathological Metastasis stage according to the 8^th^ American Joint Committee on Cancer*

*# 3 patients presented more than one metastatic localization*


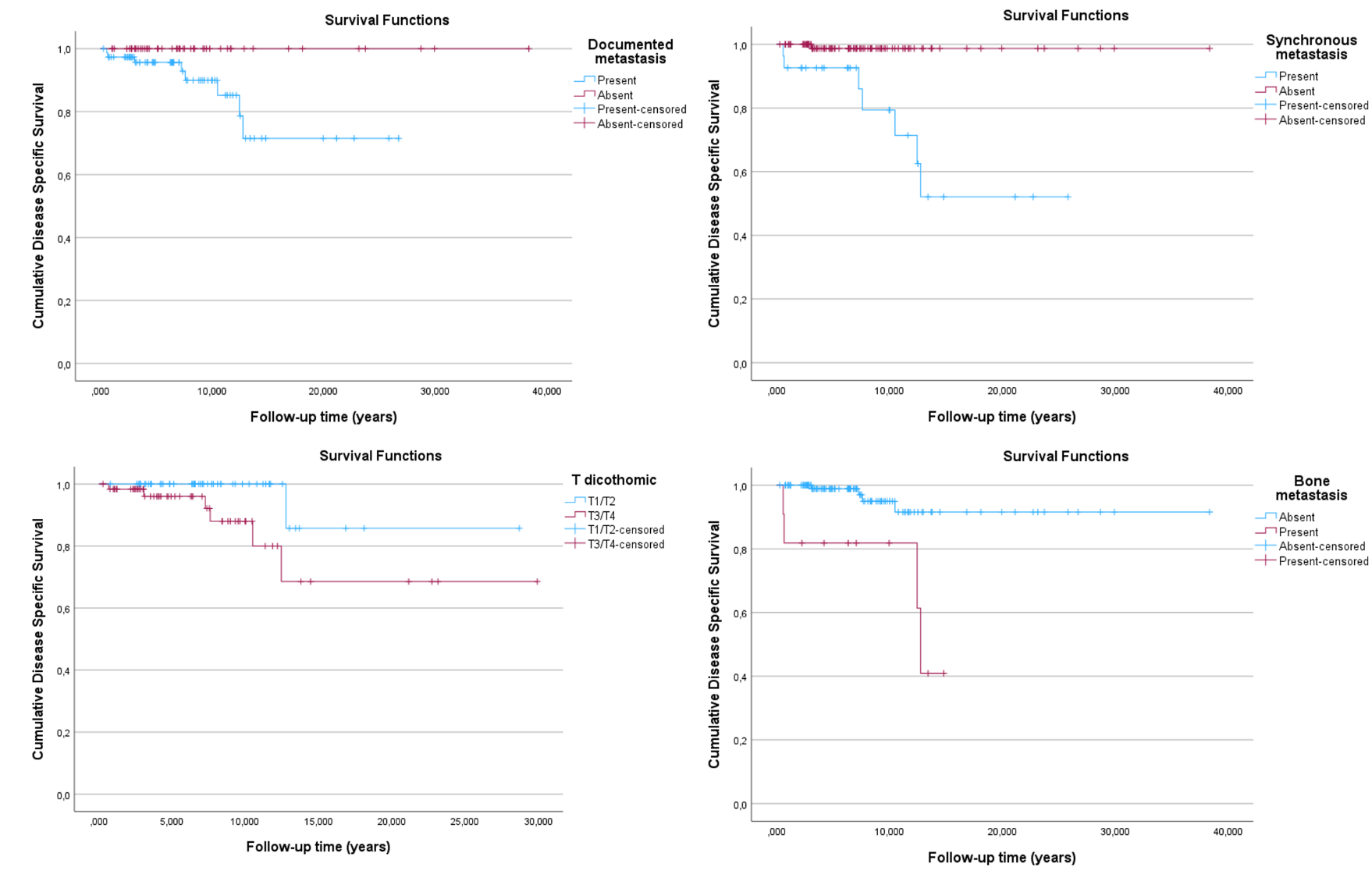


*P*<0,001

*P*=0,019

*P*=0,047

*P*<0,001

**Figure S1:** Additional Kaplan–Meier curves for DSS in the whole population
